# Achieving health equity through conversational AI: A roadmap for design and implementation of inclusive chatbots in healthcare

**DOI:** 10.1371/journal.pdig.0000492

**Published:** 2024-05-02

**Authors:** Tom Nadarzynski, Nicky Knights, Deborah Husbands, Cynthia A. Graham, Carrie D. Llewellyn, Tom Buchanan, Ian Montgomery, Damien Ridge

**Affiliations:** 1 School of Social Sciences, University of Westminster, London, United Kingdom; 2 Kinsey Institute and Department of Gender Studies, Indiana University, Bloomington, United States of America; 3 Brighton and Sussex Medical School, University of Sussex, Brighton, United Kingdom; 4 Positive East, London, United Kingdom; Washington University in Saint Louis, UNITED STATES

## Abstract

**Background:**

The rapid evolution of conversational and generative artificial intelligence (AI) has led to the increased deployment of AI tools in healthcare settings. While these conversational AI tools promise efficiency and expanded access to healthcare services, there are growing concerns ethically, practically and in terms of inclusivity. This study aimed to identify activities which reduce bias in conversational AI and make their designs and implementation more equitable.

**Methods:**

A qualitative research approach was employed to develop an analytical framework based on the content analysis of 17 guidelines about AI use in clinical settings. A stakeholder consultation was subsequently conducted with a total of 33 ethnically diverse community members, AI designers, industry experts and relevant health professionals to further develop a roadmap for equitable design and implementation of conversational AI in healthcare. Framework analysis was conducted on the interview data.

**Results:**

A 10-stage roadmap was developed to outline activities relevant to equitable conversational AI design and implementation phases: 1) Conception and planning, 2) Diversity and collaboration, 3) Preliminary research, 4) Co-production, 5) Safety measures, 6) Preliminary testing, 7) Healthcare integration, 8) Service evaluation and auditing, 9) Maintenance, and 10) Termination.

**Discussion:**

We have made specific recommendations to increase conversational AI’s equity as part of healthcare services. These emphasise the importance of a collaborative approach and the involvement of patient groups in navigating the rapid evolution of conversational AI technologies. Further research must assess the impact of recommended activities on chatbots’ fairness and their ability to reduce health inequalities.

## Author summary

Artificial intelligence (AI) systems like conversational chatbots are increasingly being used in healthcare settings. However, there are concerns that these AI tools may perpetuate biases and worsen health inequalities if they are not designed and implemented carefully with diverse populations in mind. Our study aimed to develop a roadmap to help ensure that AI chatbots for healthcare are created and deployed in an equitable and inclusive way. We reviewed existing guidelines on ethical AI use in healthcare and interviewed 33 stakeholders including community members, healthcare professionals, and AI developers. Based on this, we outlined a 10-stage roadmap covering key activities such as involving underrepresented communities in co-production, conducting research on AI biases, implementing safety protocols, evaluating real-world effectiveness, and planning for chatbot maintenance or termination while minimising disruption to care. The roadmap emphasises the importance of close collaboration between chatbot creators, healthcare providers, policymakers, and the communities being served. It provides a checklist of equity considerations at each stage of the chatbot lifecycle. Following this roadmap can help mitigate unfair biases, increase acceptability among diverse patient groups, and harness the potential of conversational AI to improve healthcare access and reduce inequalities.

## Introduction

Artificial Intelligence (AI) has established a substantial footprint in the healthcare sector, offering promising avenues for improving patient outcomes and optimising clinical workflows. AI encompasses various technologies, such as machine learning and natural language processing, and finds applications in diverse areas, including disease diagnosis [1], drug discovery [2], medical imaging [3], and electronic health record management [4]. Despite its potential for increasing healthcare efficiency and reducing costs, AI implementation is fraught with challenges, including data security and governance, ethical concerns, and the need for diverse training datasets [5].

A specific subset of AI in healthcare is patient-facing conversational AI agents and chatbots, which directly interact with patients to perform tasks ranging from symptom self-diagnosis and treatment recommendations to medication management [6]. These include various modalities such as text-based chatbots [7], voice assistants [8], and wearable devices [9]. While these technologies offer potential benefits regarding increased access and improved health outcomes, concerns about their effectiveness, safety, and potential to exacerbate health inequalities have been raised [10]. Within this landscape, conversational AI have emerged to enhance patient engagement and facilitate communication between patients and healthcare providers [11]. They offer capabilities such as scheduling appointments, giving medication reminders, and providing mental health support. However, implementing conversational AI is not without risks, with ongoing concerns about data security, accuracy, and the potential to respond in ways that do not recognise the needs of users from minoritised communities [12].

Several frameworks exist for evaluating and implementing new technologies in healthcare settings, including the Proctor model [13], the Consolidated Framework for Implementation Research (CFIR) [14], and the Reach, Effectiveness, Adoption, Implementation, and Maintenance (RE-AIM) framework [15]. However, these frameworks lack specific guidance for handling the unique challenges associated with conversational AI technologies. Despite the advances, there is an acute lack of guidelines for ensuring that conversational AI tools are designed and implemented equitably. Current frameworks provide some direction but are insufficient in addressing activities aimed at ensuring greater equity, diversity, and inclusion [16]. For example, a roadmap for responsible machine learning for healthcare recommends ethicist engagement for potential bias correction but does not actively suggest an effective co-involvement of diverse patient groups [17]. Given these gaps and challenges, there is a need for a tailored framework for the design and implementation of conversational AI that focuses on eliminating biases and ensuring cultural competence, referring to their ability to engage with users in a manner that is informed by and sensitive to diverse cultural contexts, including language, customs, and social norms [18]. Such conversational AI tools can tailor interactions to meet the specific cultural needs and expectations of individuals, thereby improving communication effectiveness and user satisfaction.

Thus, a conceptual framework can help identify potential biases, safety issues and gaps in conversational AI performance for different groups early on. Centring equity as a priority aligns with ethical goals around fairness and avoiding harm [19]. It could also offer an outline of structured best practices for equitable conversational AI design and deployment promoting transparency, accountability and monitoring [20]. As such, our study aims to identify potential activities that could contribute to greater equity in healthcare through the implementation of conversational AI and to develop a comprehensive framework guiding developers on equity, diversity, and inclusion issues.

## Methods

### Design

The study utilised a qualitative approach to investigate the implementation of equitable conversational AI in healthcare services. We began with a content analysis approach [21] to review policies and guidelines related to deploying conversational AI in healthcare services. We aimed to identify existing recommendations around equity and fairness for their design and implementation. Based on that scoping exercise, we drafted a conceptual framework proposing an equitable implementation process, which we used as a basis for subsequent stakeholder consultation. We then conducted semi-structured interviews to gather feedback from stakeholders on the proposed framework, which we then used to refine and finalise the roadmap. The interview data were analysed using a framework analysis approach [22,23] to inform the development of an implementation roadmap for equitable conversational AI in healthcare.

The Westminster Research Ethics Committee granted ethical approval [ref: ETH2122-0782]. An information sheet was given to each participant, and they provided written consent before each interview.

### Development of the conceptual framework

In July 2022, we conducted a scoping exercise on Pubmed, in which we searched the database using specific keywords such as “artificial Intelligence”, “AI”, “chatbot”, “conversational assistant”, “guide”, “framework”, “recommendation”, “health”, “healthcare”, and “implementation” to find guidelines related to the implementation of AI-led chatbots in healthcare services. This scoping exercise aimed to identify policies that covered technical, legal, ethical, and procedural aspects related to the design and implementation of patient-facing conversational AI agents into healthcare systems, specifically emphasising equity, diversity and inclusion. As such, we included publications specifically concerned with the design and deployment of AI in the healthcare context. We excluded articles that did not contain a comprehensive set of rules or guidelines for conversational AI designers, or which were not presented as policy statements. There were no geographical or time restrictions, but only publications in English were included. Our search focused on identifying conversational AI -specific guidelines and policy papers that looked broadly at the implementation of conversational AI systems and those broadly exploring AI use within the UK National Health Service context.

After scanning the titles and abstracts of the 220 potential papers identified in our search on Pubmed, we found only three that met the inclusion criteria (e.g., policy guideline or a technical framework aimed at designing and implementing conversational AI or chatbot for healthcare). Since policy papers were not commonly published in scientific journals, we expanded our search to include grey literature by conducting Google Scholar searches and consulting key stakeholders/institutions that focus on AI implementation in healthcare (i.e. The World Health Organisation and UK National Health Service). Through this process, we identified 17 policy articles related to either the ethics, governance, or evaluation of conversational AI systems for healthcare (S1 Table).

The contents of the identified articles were read to extract recommendations related to the design and implementation of conversational AI, with an emphasis on increasing fairness and equity. This involved a review of each article’s content to identify sections that contained actionable recommendations. Any recommendations that were not directly relevant to conversational AI systems (e.g. wearable devices and sensors) were not included due to their limited relevance. The extracted content was organised into an Excel file for further categorisation and the management of various domains. Recommendations were then coded based on their content. This coding process involved assigning each recommendation to categories such as safety, user involvement, and conversational AI development. Coding allowed for the structuring of data into meaningful themes. The coded domains were then grouped into discrete sections corresponding to different deployment phases of conversational AI: design, pre-implementation, implementation, and post-implementation. This phase-wise organisation helped us understand how recommendations were applied to each stage of conversational AI deployment. This resulted in 50 unique entries (S2 Table) corresponding to conversational AI design and development concerning their equity, diversity and inclusion practices. These 50 entries were then used for subsequent stakeholder consultation to better understand unique recommendations and actions for conversational AI design and development.

### Stakeholder consultation

We used a stakeholder consultation approach to gather input and feedback on our proposed framework from various professionals and community members who had an interest in conversational AI implementation [24]. Here, we aimed to gather diverse perspectives and insights about the 50 entries as a basis for a roadmap to inform equitable approaches when designing and implementing conversational AI systems. The range of stakeholders’ perspectives was analysed to enhance the quality and relevance of our conceptual framework.

Between September 2022 and February 2023, we conducted 33 audio-recorded semi-structured interviews via MS Teams to gather formative feedback on our provisional concepts and domains for constructing a roadmap for the equitable implementation of conversational AI in healthcare. We interviewed a range of AI developers, designers, healthcare professionals, health educators, and community members, about their experiences designing and implementing conversational AI in the context of activities related to equity, diversity, inclusion, and bias identification/removal. Our recruitment was based on the search of online databases for private companies and charities that have engaged in the development of conversational AI systems such as conversational AI or virtual agents (i.e. NHS AI Lab, Google Scholar and Arvix). We also recruited those in the healthcare sector and academia who had conducted research projects that had developed conversational AI. We used direct engagement via email, tailored to each individual, to request participation in the study. We also used social media advertising (i.e. Twitter and Linked In) for anyone interested in equitable and fair AI. Our combined convenience and snowball sampling approach aimed to recruit a wide range of participants with expertise in conversational AI, health equity, and the voices of people from minoritised communities able to advise on the process of co-production and co-design. We recruited stakeholders from Asia, Africa, Europe, and the Americas to reduce our selection bias. Participants provided feedback on the 50 unique entries and further discussed activities to ensure equity in conversational AI systems for healthcare. Two researchers (TN and NK) conducted all interviews online using Microsoft Teams with a live transcription function that produced textual data.

### Data analysis

We used a framework analysis approach as it allowed us to analyse data within the context of our conceptual framework based on the 50 unique entries identified in the literature search. This method is particularly suitable for applied policy research with specific questions, a limited time frame, a pre-designed sample, and a priori issues. Framework analysis involves systematically organising and analysing data using a predetermined set of themes or categories [22]. In our study, we used the 50 entries developed through a content review of policies and guidelines to understand further the activities and processes to increase conversational AI equity and fairness. We familiarised ourselves with the data by checking the Teams audio transcription for accuracy and reading interview transcripts. We then entered all transcripts into NVIVO software for subsequent analysis, with the 50 domains used as priori codes for data organisation and categorisation. Next, we indexed and charted all codes into sections corresponding to the chatbot deployment’s phases relevant to the design, pre-implementation, implementation, and post-implementation stages. We mapped our findings to develop an equitable health conversational AI implementation roadmap. Two researchers (TN and NK) conducted the analysis independently to enhance the data analysis’s transparency and reproducibility and to ensure the method’s credibility and rigour. Finally, we used stakeholder feedback towards the end of data collection to refine our roadmap and agree on the final version amongst those involved in data analysis and interpretation. We engage a public and patient involvement group throughout the process of developing the roadmap to reduce any potential bias.

## Public and patient involvement

The research process integrally involved a Public and Patient Involvement (PPI) group at key stages, particularly before finalising the research outcomes [25]. This engagement ensured that the analysis reflected real-world perspectives and addressed relevant concerns. The PPI group, comprising a diverse cohort of six public members from minoritised ethnic communities, actively reviewed and validated the study’s findings during seven structured meetings. They contributed to various aspects of the research, from data analysis to contributions to the final version of the roadmap. Their insights were instrumental in refining the various stages of the proposed roadmap, ensuring that the recommendations were evidence-based and aligned with the experiential knowledge of those affected by and involved in healthcare delivery via conversational AI.

## Results

Data were gathered from 33 interviews with key stakeholders from diverse backgrounds in terms of sex, ethnicity and sexual orientation. There were 10 community members and 23 industry experts and healthcare professionals (S3 Table for demographic information).

The analysis revealed two significant phases: “*Co-design and co-development*” and “*Healthcare system implementation*” in the roadmap (Table 1), each corresponding to specific stages: 1) Conception and planning, 2) Diversity and collaboration, 3) Preliminary research, 4) Co-production, 5) Safety measures, 6) Preliminary testing, 7) Healthcare integration, 8) Service evaluation and auditing, 9) Maintenance, and 10) Termination. Each stage outlines recommendations and activities aimed at achieving equity in conversational AI (Table 2).

**Table 1:** Equitable health chatbot implementation roadmap.

DEVELOPMENT AND IMPLEMENTATION OF EQUITABLE CONVERSATIONAL AI IN HEALTHCARE
**Co-design and co-development phase**
**1. Conception and planning**
○ Identify existing health disparities and explain how conversational AI can address them ○ Define and set behavioural and health outcomes that the conversational AI is aiming to influence ○ Define conversational AI roles and tasks aimed at reducing health inequalities ○ Define intended users of conversational AI and their characteristics (take into account aspects of marginalisation and intersectionality) ○ Define the underlying behaviour change frameworks and potential mechanisms of action on which conversational AI operates
**2. Diversity and collaboration**
○ Ensure diversity in the design and implementation team throughout the project ○ Involve various stakeholders (end users and healthcare professionals) from diverse communities in the conceptualisation and development of the conversational AI tool ○ Involve community champions and peer support groups in public and patient engagement activities ○ Adopt a user-centred and culturally sensitive approach to reduce bias
**3. Preliminary research**
○ Review evidence base for conversational AI tools concerning their acceptability, uptake and reach ○ Review existing conversational AI tools or product designs that could be adopted ○ Review ethical guidelines on the use of conversational AI in healthcare for diverse communities ○ Review and identify high-quality AI training datasets from diverse communities ○ Review and identify accessible patient-facing IT systems for wider reach in underserved communities ○ Identify barriers to engagement with conversational AI specifically in minoritised communities
**4. Co-production**
○ Co-develop and optimise the content of the conversational AI tool (i.e. knowledge base and language) for comprehension, readability and interactivity ○ Ensure language understanding, response accuracy and impartiality through user testing activities ○ Enable multiple language translation for culturally and linguistically diverse communities ○ Ensure the ease of use for those with lower literacy or disability ○ Augment conversational AI with human-like responses and characteristics in a culturally sensitive manner ○ Ensure conversational AI transparency and its ability to present itself to people from diverse communities
**5. Safety measures**
○ Address ethical issues and prevent harm from inaccurate or unreliable responses from conversational AI ○ Develop safeguarding measures to protect vulnerable users ○ Enable a human contact pathway for further support and assistance ○ Protect user privacy when collecting sensitive personal information, considering data storage and access ○ Report and manage adverse events and unintended consequences, including complaint management
**6. Preliminary testing**
○ Proof-of-concept testing with diverse communities ○ Adress aspects related to user hesitancy or disengagement with conversational AI ○ Gather and incorporate user feedback ○ Measure the impact of conversational AI on behavioural and health outcomes within a diverse sample
**Healthcare system implementation phase**
**7. Healthcare integration**
○ Understand the healthcare system and clinical pathways for the supplementary role of conversational AI ○ Define an adequate level of governance and gain regulatory requirements/approvals ○ Clarify outstanding legal and licencing matters (i.e. intellectual property and third-party involvement) ○ Define accountability for system failure, glitches and malfunctions ○ Define regional variations in healthcare delivery and user characteristics for AI integration and tailoring ○ Define scalability and generalisability of conversational AI across the healthcare system ○ Develop staff training materials and resources on how to administer and supervise conversational AI
**8. Evaluation and auditing**
○ Conduct a feasibility study exploring usage, satisfaction and confidence in conversational AI ○ Assess uptake and engagement in marginalised communities across healthcare ○ Set up regular auditing with multiple outcomes and various methodological designs ○ Assess the sustainability of conversational AI within healthcare and community settings (e.g. cost-effectiveness)
**9. Maintenance**
○ Implement regular system updates and technical improvements ○ Understand the impact of conversational AI on the healthcare environment, service delivery, and clinical team workloads ○ Raise awareness and promote conversational AI in diverse communities
**10. Termination**
○ Design termination procedure for conversational AI within the healthcare environment ○ Assess the impact of AI termination in marginalised communities ○ Ensure the continuation of patient care through the identification of alternative services ○ Monitor and evaluate the impact of AI termination on healthcare service delivery

**Table 2:** Roadmap domain and corresponding quotes.

Domain	Exemplary quotes
**Conception and planning** **Diversity and collaboration** **Preliminary research** **Co-production** **Safety measures** **Preliminary testing** **Healthcare integration** **Evaluation and auditing** **Maintenance** **Termination**	**P19 (clinician**) ‘If the NHS is your target customer, what do they expect when it comes to buying the services? Are they buying something based on outputs or outcomes? They want to see much like you would with an ad tech or a Facebook campaign. Are they looking to see impressions or reach or are they looking to see behaviour change? They want to see uptake of counselling, screening services. If that’s what they’re looking for, that’s what you wanted to design your digital solution towards. [...] I think it probably needs to be given at the moment the way that services are commissioned, it needs to be given to each trust, and particularly because those local trusts will be more familiar with the local needs assessments of their communities’ **P12 (developer**) ‘We did some preliminary work, try to understand why people are anti-vaccine. So one thing we did is we use social media data. So we collect, you know, like people’s opinions... analysis, you know from social media. And then we try to align that to health belief models and some other models and to analyse why…You know we’re developing conversational agents for vaccine promotion’ **P18 (developer)** ‘So I think …how we build our company, it’s been specifically around diversity as well and not as a sort of corporate buzzword or anything … we have a fairly equal mix of male and female engineers, which is quite hard to do in this field, right? But then we have ethnicity diversity in terms of background… we’ve got a lot of everything from Asian to European cultural. We’re ...don’t see everything represented, but we are as we’re growing specifically looking for these things, because we believe what we build is for everybody. And the only way to deal with unconscious bias is by having people from different backgrounds different education, different ethnicities, different everything. Because otherwise you will be blind with the best intentions. I’m the stereotypical white male in it. It doesn’t get much more stereotypical, right?’ **P18 (community member)** ‘…targeting my own group, like Hispanic people like I wouldn’t like that, to be honest…kind of phrasing it like we’re giving you tailored advice according to your ethnic background, I wouldn’t like that. But it’s completely different if you’re just like ticking boxes. So, if at the beginning of the conversation with the chatbot, he asks me….asks for kind of demographics and it includes my ethnic background. I wouldn’t care. Does that difference make sense to you?’ **P39 (community member)** ‘So I’m Muslim. In Islam it’s … There’s a difference of opinion because in Islam, I don’t … I do think I have like decent knowledge. God has given us the brains we must use the brain when necessary and I don’t see any harm in that. No one’s being hurt. In fact, people are being helped. So in that case, I believe the religion would not condone this. It would in fact encourage it. But there’s other people who are bit less educated, who twist their religion. They think God is God is the creator, he’s the one. Why should we replace humans with artificial intelligence? But God has created the brains for people to create the artificial intelligence. You should be able to use your brain to the best of the capacity. If you have the resources, use it’ **P21 (clinician)** ‘I like the preliminary research section. That’s really nice because you’re building on existing evidence which is something that people sometimes use as almost like a get out clause for addressing health disparity issues. So in our project, for example, we say you need to do your own scoping around where health inequalities may already exist in your use case, and then use that information to then inform your decisions for algorithm development or data selection’ **P22 (clinician) ‘**you see this a lot generally from tech designers, chatbot designers where they don’t, they …often forget to put in the preliminary research is what is the research for the medical condition you are trying to solve. And is there any research that technology maybe not chatbot technology, but technology has been used to solve that issue. So, for example, we use chatbot technology to surface mental health support. The chatbot technology is new but online CBT has been around for 10-15 years…. So we can talk about the evidence for online CBT to say going online is plausible and commutable.... Let’s try adding a chatbot to that. I think a lot of people tend to miss that - they focus so much on proving the chatbot’ **P6 (developer)** ‘I get worried with nuanced health information and using that for Google for translation purposes. We actually are publishing an article totally unrelated about Spanish language vaccine misinformation and how Google Translate actually can promote misinformation because they translate it incorrectly and there’s a lot of real nuances. I think, especially if you’re making a chat bot for a specific group of people that uses the language that they use and that the slang terms that they use, I personally would not trust Google Translate. I would have someone in the specific community that I’m working in, translate them’ **P36 (community member)** ’ the pictures they put in, the colour as well. I know it’s something little, but like if I see a chatbot with brown skin I’ll be like OK. I don’t if that if that makes any sense, you know? Like on chats on WhatsApp, there’s the emoji they made it of different colour. Now if I see one of that, it’s more appealing to find something of my skin and I’ll be like, OK or maybe one that has someone you know, the Muslim burka. It’s kind of appealing. I think it’s it shows inclusiveness from my point of view’ **P21 (clinician)** ‘I would love to see something like somewhere… somewhere in this list around public transparency. I don’t know if you have that but staying accountable to the public around like safety incidents. Or you know just bad things that happen essentially... So, for medical devices, um, in the US for example, if you’re registered on the FDA, you have an adverse event, it’s actually captured on a publicly available uh public facing database called Maude …I think is really good practice is to have some public facing accountability around things that may have gone wrong, and what you’ve done about it. I think there’s something around public transparency which would be really good here’ **P2 (clinician)** ‘People putting in, you know, for example, details about sexual partners. There needs to be no patient identifiable data. Any kind of any interactions in the chat bot that have been evaluated need to have any kind of patient ID taken out and unless they work in the department then you know you can obviously kind of link it up, but I think it’s more information governance than the use of the that the use of the data once patients have put in, you know what they’re there for. And if you were to go down the route of asking about risk for example, so you’re asking about sexual behaviours. Even things like their IP address need to be masked, because obviously they’re telling you they have sex with guys and they’re married’ **P17 (developer)** ‘but there is definitely some back and forth between the different steps as well and just... Yeah. I think that would just help to illustrate the overall process, because it’s not, you know, it’s not just a linear process like that. There’s a lot of steps that, you know, maybe involve going back to speak to the user groups or the professionals again. And one of the other things, that was useful for us earlier on… specifically usability testing with users, with the initial prototype, because one of the most frustrating things that we found when talking to people was technical errors that were coming up and immediately putting people off using the chatbot... Altogether... so the sooner you can iron out those problems, the better’. **P23 (community member)** ‘I also think that you know nowadays we can … book restaurants just saying okay Google, you know, book me a restaurant somewhere. Why can’t we do that with sexual health... and it’s used like, obviously there are things to consider that are very much related to confidentiality, but why can’t we have a chatbot that can actually give me the service I want? Instead of frustrating me by telling me that I have to access this link and then press these buttons, you know, put it, put it there, you know, make it easy for the, for, for the population for everyone’ **P22 (clinician)** ‘So, one of the big challenges of being adopted in the NHS is we did an accessibility and adoption report as part of our last study and the majority of staff when we asked them what do you think AI is? They either thought it was like the chatbot that you talked to when you’re trying to do your online banking and you spell out the password and it spells you back a completely different word. Or they thought it was like the Terminator, like there was no in between… no middle ground, and so there’s a lot of hearts and mind stuff to be done with clinicians because ultimately if you’ve got a patient facing technology or a clinician controlled technology, you have to get the frontline clinicians to believe in your product or they won’t implement it’. **P10 (clinician)** ’Evaluation is also really challenging to be honest with you. How one goes about determining whether a chatbot is good or bad is not straightforward. There is, first of all, like clinicians aren’t themselves accurate. Tests that clinicians take are like a really artificial environment... patient outcomes are probably the best measure of success, but they require a lot of longitudinal data. And it has a lot of selection bias attached to it. So yeah, evaluation is really challenging, and I would say probably the best approach is like a multi-pronged approach over a long period of time. It’s something that, you know, I think is really, really hard because the level of investment that is required is very high, and the ROI on it is questionable, interestingly enough, so I think it’s something, you know, to really think carefully about in terms of your strategy there.’ **P3 (academic)** ‘The way they would be evaluated is very different. So, it depends on what the chatbot is doing, if it’s, for example, diagnosing a disease. You would do a, you know, like an accuracy type study, if it was delivering therapy like there’s lots of mental health chatbots that are delivering cognitive behavioural therapy, talking therapies. You’ll be doing an effectiveness study.’ **P19 (clinician)** ‘Because I think, um, actually to implement a tool that is, uh, safe, efficient, and beneficial costs probably more than many people anticipate, and so does the design, development and implementation process of it. And I don’t think necessarily that you will see any efficiencies, outback end of implementing something like a chatbot for some time’ **P20 (clinician)** ‘if the chatbot needs changing, then has the... if it’s interoperable with other systems that have the risk of all these other systems falling down. So, any change needs to be done within a test system that can integrate with the test system of the other health informatics systems that are being used. So, we, we’ve recently stopped using a digital provider for our electronic or our digitally sent out letters because they can’t provide a test system’ **P20 (clinician)** ‘Removing the chatbot from the site. If something critical were to happen, we would find that it was causing more harm than good. Removing it would be trivial. Yeah. Also, I think it is within the scope of their work because essentially, how we would deliver it would be to give them instructions on how to add it to their website, which would be quite a simple process. So I think it would be on them to reverse those instructions.’ **P1 (technical expert)** ‘You need a long leading time for doing switched off. It needs communication with the department and the patient base that are using it about why it’s being turned off and when it’s being turned off. You need to leave a similar landing page or similar area or website around signposting for different things so that people can still access information online. And there needs to work within the department around trying to figure out the unmet need once it goes offline and trying to kind of compensate by either more staff or changing pathways or changing the websites or changing the other access points into the service for advice or so I think it needs a long ...well three month plus warnings being switched off and then people need to know that switched off because people may rely on it more than once or twice for advice. And it needs to be replaced with something obviously, either a human or another chatbot to replace it.’

### Stage 1: Conception and planning

According to the interviewed stakeholders, when designing conversational AI with equity in mind, it is important to identify public health disparities and determine how conversational AI can help mitigate them. Conversational AI should be designed to address specific illnesses or conditions that disproportionately affect minoritised populations due to factors such as age, ethnicity, religion, sex, gender identity, sexual orientation, socioeconomic status, or disability. A specific ‘needs assessment’ should be first conducted to recognise health disparities and identify benefits offered by conversational AI that cannot be easily achieved through in-person services. For instance, a conversational AI tool could be developed to improve access to mental health services for rural communities that may experience greater geographical barriers to accessing healthcare. Similarly, conversational AI could offer non-judgmental advice and signposting to relevant sexual health screening services for members of religious communities, who may be reluctant to discuss symptoms or screening options because of the stigma around sexually transmitted infections (STIs). As part of that needs assessment, designers should define and set behavioural and health outcomes that conversational AI is aiming to influence or change. Setting these outcomes from the beginning would enable designers to later evaluate the conversational AI effectiveness in reducing health inequalities and inform any necessary adjustments. For example, conversational AI could be designed to increase screening attendance rates among low-income patients by providing appointment booking and reminder systems. By setting specific targets, such as a 20% increase in appointment attendance among low-income patients, conversational AI’s success in achieving its goals can be quantified and evaluated.

Also, participants noted that it is essential to define the conversational AI role in clinical or administrative tasks to clarify its scope and responsibilities. Conversational AI can serve various functions, such as providing health education, facilitating appointment scheduling, or assisting in medication adherence. By clearly delineating the purpose of conversational AI, designers can optimise its functionality to ensure it addresses specific aspects of health inequalities effectively. For example, conversational AI designed to reduce disparities in cancer screening rates may focus on providing tailored educational content that is relevant to each user. Understanding the intended users and their characteristics involves considering aspects of marginalisation and intersectionality to develop conversational AI that is sensitive to the diverse needs and experiences of its users. By considering demographic and social factors, designers should ensure that conversational AI is accessible and engaging for users from varied underserved backgrounds. For instance, according to one stakeholder, conversational AI addressing prenatal care disparities among low-income, ethnic minority women may incorporate culturally relevant information to create a more inclusive user experience. Additionally, two stakeholders mentioned the importance of understanding the underlying behaviour change framework or the theoretical grounding for conversational AI to predict its mechanisms of action as well as to guide the development process and ensure that conversational AI tools are evidence-based and effective. By grounding conversational AI in established behaviour change theories, it was thought that designers could increase the likelihood that it would successfully influence users’ behaviours and contribute to improved health outcomes.

### Stage 2: Diversity and collaboration

All stakeholders agreed that conversational AI tools should be designed with input from the communities they are intended to serve. The roadmap highlights the importance of diversity in both conversational AI design teams and community input to reduce the potential for bias and ensure high acceptability, optimal uptake, and overall user satisfaction. As such, accommodating a wide range of perspectives is crucial at the initial stages of development, especially during the conception and planning of conversational AI. For example, despite good intentions, developers might inadvertently introduce their unconscious biases into the conversational AI tools’ language, content, and expressions. Therefore, contributions from a diverse group can help to identify potential ‘blind spots’ and counteract this effect. Users from specific ethnic minority communities may face more obstacles due to lower levels of health and digital literacy, and a diverse design and implementation team can ensure conversational AI is accessible and usable for all, especially the most disadvantaged groups. It is therefore considered critical to involve patient and public involvement and engagement (PPIE) groups in the design of conversational AI to ensure their significant influence on the process. Establishing ties with relevant community groups can help better cater to the needs of these patients.

There was a view that conversational AI design teams must strive to comprehend the genuine needs and preferences of specific patient groups, ensuring that the technology is ‘culturally competent’. For example, topics such as sexual and mental health often carry high levels of stigma and may evoke feelings of shame or embarrassment in some ethnically minoritised groups. The stakeholders recommended that conversational AI designs should create a comfortable environment for discussing sensitive issues among people of different cultural and ethnic backgrounds. For instance, if conversational AI is developed to tackle sexual health disparities, users’ preferences for content addressing feelings of stigma and shame should be incorporated. On the other hand, labelling some social groups as more ‘*at risk’* could be considered offensive, making individuals feel unfairly targeted by the conversational AI, thereby perpetuating stereotypes contributing to health inequalities. As such, culturally competent conversational AI can be designed to recognise diverse cultural beliefs and values, however, they work best when they use neutral, medically accurate content focusing on health-related behaviours over culturally nuanced language. Neutral language minimises the operation of stigma and enhances understanding of complex health matters.

Nevertheless, it was thought that if users do not believe that a conversational AI tool is relevant to them or capable of addressing their unique health goals, they are less inclined to interact with it. To make users feel that thought has been put into how conversational AI can best meet their individual needs, conversational AI tools should be developed with contributions from people within their own communities. Developers should also carefully consider the ’message’ they intend to convey to their target audience before users engage with conversational AI. For instance, certain minority groups may regard conversational AI with suspicion, mistrust and scepticism due to historical racism, experiences of medical exploitation, ethical concerns regarding the technology, or religious beliefs. Involving community champions or leaders could play a crucial role in understanding conversational AI acceptability and addressing potential conflicts with specific social and cultural factors.

While conversational AI developers do not always collaborate with health professionals in designing and developing conversational AI, it can be beneficial to work closely with frontline clinicians and clinical safety officers from diverse cultural backgrounds or who have expertise working with the target patient group. Collaborating with health professionals can ensure the accuracy and relevance of medical content and the consideration of safety issues salient for specific social groups. Patient advocacy groups and professional bodies can help identify barriers and forms of bias that might go unnoticed. Health professionals can also guide how conversational AI best integrates with existing care pathways and services. Involving them in the design and implementation phases can enhance their knowledge and understanding of how conversational AI operates and demonstrate these technologies’ potential benefits, such as reducing workload and improving clinical outcomes.

### Stage 3: Preliminary research

Many stakeholders agreed that developers creating a conversational AI tool should explore existing conversational AI interventions and services before creating anything new. In some cases, expanding the content of an already successful and evidence-backed conversational AI may be more equitable and cost-effective, especially if it aligns with the targeted health concern. If an appropriate intervention is unavailable, a feasibility estimation and technical exploration are required to understand the impact of various conversational AI designs on non-discriminatory delivery. For example, selecting an appropriate platform for chatbot-patient interaction requires careful consideration and examination. Conversational AI hosted on health provider websites, such as the NHS, are generally more trusted by users. Those hosted on non-NHS platforms may impose limitations on the type of content or delivery they permit, affecting conversational AI impartiality. Similarly, developers should consider chatbot technical complexities to meet user needs, regarding certain age groups, digital literacy or cognitive abilities. Access to technology is likely to determine the success of conversational AI interventions for marginalised and underserved groups; thus, the design team needs to understand how to produce an intervention that is most accessible to users who do not own devices such as smartphones or tablets.

Before any training of conversational AI modules takes place, the conversational AI design teams can identify high-quality training datasets that incorporate diverse communities and populations. If datasets fail to represent the intended patient group, biases could become further ingrained in healthcare, negatively affecting these groups. Ongoing research on biases in AI medical devices includes conversational AI tools, their potential impact, and mitigation strategies that conversational AI designers should review and adopt. Developers must stay informed and follow AI and digital health guidelines for health interventions before conversational AI development occurs.

Preliminary psychosocial and behavioural research should allow designers to understand conversational AI desirability and acceptability as well as to identify barriers to engagement amongst users from ethnically and linguistically diverse communities. ‘AI hesitancy’ due to unfamiliarity with the technology or fears around confidentiality may be more relevant to specific groups and must be identified and addressed accordingly. The reluctance to disclose relevant health-related information to chatbots by people from minoritised groups may indicate hesitancy and require further assessment of feasibility. If there is no pre-existing evidence on acceptability, mixed-methods research should be conducted at this stage to inform the development of the conversational AI intervention and ensure a user-centred and community-centred approach.

### Stage 4: Co-production

Once preliminary research is concluded, involving specific patient and public groups in conversational AI design and development is beneficial. This is especially important for ethnic minority communities who may not have English as their first language or may have low English literacy levels. For example, conversational AI tools should be scripted to understand and accurately respond to slang, colloquialisms, and incorrect grammar and spelling. It is imperative to have reliable translation capabilities, especially if the conversational AI tool is intended for ethnic minority communities, migrants or refugees. Users should be able to access conversational AI content in their native language to optimise engagement and maximise acceptability. As such, developers need to involve native speakers in the co-production and translation of scripts to ensure accuracy and promote clarity and comprehension for linguistically diverse users. Interviewed stakeholders emphasised that the co-production of conversational AI content and appearance should involve people of diverse social, cultural and religious backgrounds to enable better design by understanding cultural complexities and social norms.

When deciding whether conversational AI should exhibit human-like qualities, involving people from diverse backgrounds is important. Users should have a say in the chatbot’s conversational styles, physical appearance, and whether or not it needs to establish a specific persona. The co-production process should involve diverse groups of users when refining the chatbot’s background information about its role and capabilities to manage user expectations. The process can also help to make conversational AI easier to use and more accessible, especially for users with physical health problems or disabilities. Alternative modes of delivery, such as a voice-activated conversational AI should be considered to aid users with visual impairment or physical difficulties. Similarly, any websites, patient information forms and conversational AI instructions should be co-produced with diverse groups.

### Stage 5: Safety measures

When developing conversational AI that offers medical and health advice, it is crucial to consider ethical considerations to prevent any harm or unintended consequences for users. Ensuring the utmost accuracy of the information provided by conversational AI is imperative, whether it’s a rule-based conversational AI or a large language model that generates text. Incorrect medical advice or misguided guidance could have serious safety implications for users, making it necessary to establish a standardised approach for evaluating accuracy. For instance, conversational AI models may incorporate "confidence thresholds" by providing clear statements of certainty to users when their questions are unclear or there is not enough data to provide a valid and definitive response. Conversational AI developers must also ensure language accuracy for people from linguistically diverse communities, especially if they offer automated conversational AI content translation services. Regular updates of the conversational AI knowledge base ensure that the information is accurate and in line with up-to-date medical recommendations.

It is important to recognise and communicate the limitations of conversational AI and encourage users to seek professional medical advice, especially for conditions that require urgent care. Designers should implement emergency protocols to protect vulnerable users. These protocols may involve immediate contact with a health professional or trained operator, as well as directing users to helplines or websites where they can receive help and support. Conversational AI algorithms should be designed to recognise phrases that may indicate emotional crisis, self-harm or suicidal ideation so that users can be directed to appropriate resources. When designing conversational AI to address specific health issues, such as health inequalities, the user base may be more vulnerable due to various factors, such as marginalisation, socioeconomic deprivation, and refugee status. Therefore, more rigorous safety protocols should be employed, including readily available human contact pathways with quick response times, delivered in a culturally competent manner.

When implementing health conversational AI, it is crucial to determine accountability for safety breaches and unintended consequences. The responsibility may differ based on the context of the breach. For instance, the developer may be held responsible if the breach occurred due to a poor-quality algorithm or technical errors. On the other hand, if the host organisation fails to provide adequate training for staff, they might be accountable. Therefore, developers should collaborate with clinical safety officers to ensure the safe implementation of conversational AI within healthcare services. Additionally, health chatbots should offer users an easy way to report safety concerns or file complaints.

Data security breaches are a significant concern for both developers and users. The type of information that developers decide to collect from users (such as personally identifiable, demographic, behavioural or attitudinal data) or whether the conversational AI tool is meant to operate anonymously, will depend on the chatbot’s objective and the regulations of the hosting organisation. If a conversational AI is intended to be integrated with electronic patient records to facilitate smoother care pathways, this would require careful consideration of data security and privacy across both systems, while adhering to data protection laws. If chatbots collect any personally identifiable information, secure user authentication might be required to verify their identity. Developing a comprehensive privacy policy to protect user data and use appropriate firewalls and encryption measures is crucial. It’s also important to consider user privacy when sharing sensitive or risk-based information, especially if it’s stigmatised or taboo within certain cultures and could lead to significant consequences in the event of a data security breach. Additionally, if the conversational AI is hosted on other platforms, such as social media or available as a downloadable app, additional security measures may be necessary, such as intrusion detection systems or regular security audits.

### Stage 6: Preliminary testing

After developing the chatbot’s prototype, it is important to conduct proof of concept testing to ensure its equity, safety and efficacy. The stakeholders believed that any functionality, usability, and conversationality testing should involve patient groups and diverse communities in which health conversational AI is developed. Such performance testing would also help verify conversational AI content, detect technical abnormalities, and identify potential bias. If the conversational AI provides links to other organisations or booking sites, testing should ensure they work properly, including data safety measures. User feedback is crucial for optimising conversational AI performance before finalising the prototype and proceeding with implementation. For example, the emotional responses of users, such as frustration or enjoyment, should be assessed to estimate the level of engagement. This may involve a cycle of continuous testing and usability trials, with refinement and updates to the prototype. Developers may decide to conduct a series of simulations to examine the workflow and enhance user experience before conversational AI deployment.

Conversational AI designers should also conduct pilot studies with appropriate equity metrics to assess the type and level of behaviour change in the intended user group, aligned with defined health and behavioural outcomes. For equitable health conversational AI, cultural and language sensitivity testing may be required, especially if the conversational AI targets users from ethnic minority backgrounds or includes a translation feature. These studies can also demonstrate user accessibility and acceptability of the conversational AI, allowing for a better understanding of underserved social groups that are less likely to successfully engage with the conversational AI.

If users are hesitant to engage with conversational AI, developers need to consider strategies to address these issues. Further collaboration and co-production stages, in response to primary user testing, may shed light on potential reasons for user reluctance. These reasons may include a lack of understanding of conversational AI responses, specific social or cultural factors, or a preference for a different conversational AI appearance or interface. Additional consultations with the intended patient group, healthcare professionals, and community members may be necessary to find solutions. It is also essential to incorporate a qualitative research component, such as follow-up interviews, to yield valuable data and provide a deeper understanding of how conversational AI influences outcomes or the lack thereof. Developers must provide evidence that their conversational AI is not only effective and safe but also acceptable to users from culturally and linguistically diverse communities before it is integrated into healthcare services.

### Stage 7: Healthcare integration

When creating conversational AI for healthcare organisations, it was thought crucial to understand how conversational AI can be integrated into the current care pathways. Though chatbots may expand healthcare access and improve patient health outcomes, they should not be designed to function as a standalone service. Therefore, developers and healthcare providers need to work together to ensure that the chatbot’s goals align with the broader healthcare objectives of reducing health disparities, improving health outcomes, and increasing healthcare access. It is important to conduct integration testing to ensure that conversational AI data exchange is continuous and accurate, particularly when conversational AI access electronic health records, appointment booking systems, prescription, and medication support systems where patient details are stored. It is also important to identify how chatbots can fit into existing healthcare workflows and to what extent their deployment can disrupt healthcare provision and service quality. Therefore, small-scale implementation activities must be first performed to address all issues related to integration.

Developers of conversational AI tools may not be always familiar with the governance and legislative processes that are mandatory in the healthcare sector. Therefore, it is recommended that they seek guidance from regulatory bodies responsible for overseeing healthcare delivery in the specific region or country where their tool will be deployed. Collaborating with the regulatory compliance team on legal matters can ensure that chatbots not only meet all the necessary requirements but also obtain the essential approvals and certifications. The introduction of any technology into the healthcare domain demands specific accreditations and assurances to establish standards for clinical risk management related to health technology. In cases where a chatbot collects identifiable patient data and employs encryption, obtaining the essential certification is vital. For example, this may include a penetration test to simulate potential cyber-attacks. Additionally, the deployment of a conversational AI requires careful consideration of medical device licensing and regulatory approvals during the initial stages of design and implementation. It is important to note that the specific regulatory approvals required can vary based on the intended use of the conversational AI.

When incorporating conversational AI developed by external entities or individuals into healthcare services, it is important to consider additional legal and licensing agreements. The conversational AI contract should outline the procedure in case of system failure or malfunction, and determine the responsible party. Shared maintenance responsibilities between the supplier and the host, as well as issues of ownership, should be clarified. For instance, the contract should specify who is responsible for updating the system and whether it is done regularly or as needed.

Healthcare providers may have reservations about adopting AI and conversational AI technology, stemming from a lack of understanding of conversational AI and a perception of limited benefits for underserved user groups. To address these concerns, it was considered essential to provide comprehensive staff training. Equipping staff with a clear understanding of the technology is vital, as it not only enhances their ability to use it but also empowers them to assist patients effectively. Developing user manuals and a glossary of technical terms while avoiding jargon can be instrumental in facilitating this understanding. However, it is important to note that training should go beyond the mechanics of conversational AI operation. Training should include an understanding of the chatbot’s role within the broader clinical pathway. This involves not only providing information and training but also addressing any negative attitudes and beliefs. The goal is to encourage a sense of trust in the chatbot’s user-friendliness, technical reliability, accuracy, and potential positive impact on the healthcare environment. Furthermore, it is crucial to ensure that the introduction of new technology does not increase the workload of healthcare staff.

When developing a conversational AI to address a specific healthcare challenge within one region and under the management of a particular healthcare provider, it was thought important to acknowledge that the chatbot’s performance may not be equal when deployed in a different region with distinct user groups that share different social and cultural characteristics. In such cases, adaptations to the conversational AI may be required to align with the local needs and user requirements of the new region. This flexibility allows healthcare providers to make essential adjustments, such as language preferences or interface modifications. However, since individual healthcare providers typically assume ownership of their respective chatbots, scalability can pose a substantial challenge. If chatbots are to be implemented across various healthcare organisations, it may be necessary to initiate new pilot studies to ensure efficacy and user satisfaction. Achieving scalability within each healthcare provider requires effective communication among different services and a culture that fosters shared learning and the dissemination of knowledge.

### Stage 8: Service evaluation and auditing

Assessing the impact of health conversational AI tools on the healthcare sector was considered to require service evaluation and auditing, with a focus on promoting equity and addressing health inequalities. Collaborating with academic health science networks can provide valuable support to health innovators and digital transformation efforts. It can help in understanding the wider implications of conversational AI for public health outcomes. Conversational AI implementers must precisely plan their evaluation approach, considering factors such as time constraints, financial investments, and practical and ethical considerations. A thorough evaluation of chatbots is essential to assess their effectiveness in mitigating health inequalities among marginalised patient groups. The evaluation should focus on the impact of conversational AI on specific health and behavioural outcomes. The choice of evaluation method will depend on the chatbot’s type, potentially involving factors such as feasibility, patient satisfaction, behaviour change, or the influence on health outcomes in areas of illness prevention, diagnosis, treatment, or management. Monitoring the conversational AI features most frequently utilised by patients and incorporating a user feedback mechanism, such as a rating or suggestion system, can be invaluable for continuously enhancing the chatbot’s utility and effectiveness.

Patient outcomes are a reliable measure of effectiveness, but they might require collecting longitudinal data or following up with patients across various clinical pathways. When conversational AI tools operate anonymously, additional complexities may arise, and service-level metrics, such as healthcare service utilisation across various social groups, may be considered. When evaluating conversational AI aimed at reducing health inequalities, implementers may seek detailed information about patient uptake, including demographic data. However, it is essential to clearly communicate the purpose behind collecting such information, emphasising its role in enhancing service quality and improving patient satisfaction and outcomes. Conducting pre- and post-test studies using anonymous user identifiers to link data can also provide valuable insights. If initial effectiveness is demonstrated, implementers may consider comparative evaluation approaches to determine conversational AI acceptability, reach, cost-effectiveness and equity when compared to existing service modalities.

### Stage 9: Maintenance

The sustainability of health conversational AI hinges on various factors, but participants believed a keen focus on user satisfaction, especially amongst minoritised communities, helped. Additionally, alleviating the workload of healthcare staff while maintaining overall cost-effectiveness was thought important. The introduction of a high-quality, secure, and efficient conversational AI entails an initial financial investment, and a compelling case must be built to demonstrate how conversational AI can potentially lead to cost savings. However, it is essential to acknowledge that the financial benefits may not become immediately evident, given the expenses associated with the design, implementation, and evaluation of equitable conversational AI tools. Taking a holistic perspective, considering whether other advantages, such as enhanced safety and improved staff well-being, can outweigh the immediate need for cost savings was recommended by some. While projected cost-effectiveness may have been part of the initial business model discussions, a comprehensive understanding and assessment of how conversational AI functions within the clinical pathway and its potential for cost reduction in specific areas post-implementation may require some time to become apparent. The overarching aim is to balance the financial aspects with the equitable provision of healthcare services.

Regular updates were considered vital for a health conversational AI to stay current with the latest clinical, medical, and technical advancements. It is essential to establish clear guidelines and protocols for executing these updates. The responsibilities for these updates should be well-defined, whether assigned to the developer, healthcare provider, or other hosting organisation. Although the developer may provide guidance on the maintenance and operation of the conversational AI, some level of technical expertise may be necessary. Including this in the training for healthcare and IT support staff can facilitate the process. If conversational AI tools are to become a permanent part of healthcare, they should be considered a mainstream addition to health technology rather than a specialised domain. Given the significant impact conversational AI tools are likely to have on the future of healthcare, developers should proactively contribute to disseminating new knowledge and educating healthcare staff.

Maintaining and updating health chatbots is crucial, and one of the most important aspects to consider is their interoperability with other systems in the clinical pathway. Interoperability is vital because conversational AI can serve as a link within a broader healthcare technology ecosystem. Therefore, developers must ensure that any changes made to the conversational AI do not unintentionally disrupt or degrade the performance of other interconnected systems. This requires careful coordination of even minor updates to the conversational AI, to maintain harmony within the technology ecosystem as a whole. If conversational AI modifications do affect other technical systems, provisions should be in place to rapidly identify, monitor, and address any issues that arise. This may involve real-time system performance monitoring and a troubleshooting protocol to rectify issues as they arise. Regular and comprehensive testing of the conversational AI within the network of systems can also help identify potential issues before they result in significant disruptions.

To ensure the sustainability of health conversational AI tools, it is important to evaluate their impact on healthcare services and staff workload. This involves reviewing the chatbot’s initial goals, such as providing health education or increasing access to services. The main concern is whether these goals were achieved and if the conversational AI has effectively reduced staff workload, allowing them to focus on patients from marginalised communities with more complex needs. If the conversational AI does not operate optimally, it might lead to technical issues and patient complaints. Thus, regular assessments during the early stages of implementation can help gauge staff attitudes and experiences with the conversational AI, identifying and resolving emerging issues, and enhancing its long-term viability.

For conversational AI to have a substantial impact on public health, it is crucial to ensure that the conversational AI is visible to diverse communities. The conversational AI tools should be prominently featured on healthcare providers’ websites and other relevant platforms, highlighting the evidence base for its effectiveness and safety to encourage user trust. Clear information on the ethical dimensions of conversational AI should be provided, particularly for individuals from minoritised groups who may have historic healthcare trust concerns due to racism, discrimination and marginalisation. Community champions who are involved in the design phase can play a pivotal role in increasing awareness about conversational AI after its implementation. They can offer insights into the best avenues for promotion. Charities, advocacy groups and third-sector organisations supporting minoritised communities can help raise awareness and provide access to digital health technology. Therefore, addressing hesitancy towards conversational AI is essential, as inadequate conversational AI usage, if not handled carefully, could inadvertently exacerbate health inequalities.

### Stage 10: Termination

When developing a health conversational AI, participants noted that anticipating scenarios in which discontinuing the conversational AI service becomes necessary is part of their lifecycle. This may happen when the conversational AI poses a threat to patient health, fails to deliver expected benefits to the healthcare environment, or gets replaced by more advanced technology. Developers must offer host organisations clear instructions on removing conversational AI from a website or digital platform. However, in the context of equitable health conversational AI tools, there are specific considerations to keep in mind. For example, conversational AI may be deeply integrated with other systems and could function as an integral part of the patient care pathway, especially for those with more complex needs, such as people from linguistically diverse communities. As a result, removing it could have cascading effects on these interconnected systems and patient care. These dependencies must be carefully considered in advance to mitigate complications, time-consuming modifications, and potential financial implications. Depending on the chatbot’s role in healthcare, developers should establish a removal policy, especially if the conversational AI is a medical device. If termination becomes necessary, it is crucial to have a well-defined termination period. This allows users and healthcare providers to prepare for the chatbot’s unavailability after a specific date. Such advance notice enables patients and healthcare providers to make alternative arrangements and re-establish the clinical pathway without any disruption.

An appropriate disclaimer or notification of conversational AI termination should be communicated, informing them that the service will no longer exist or receive updates beyond a specific termination period. If a conversational AI tool was initially distributed as a downloadable app, it is crucial to guide users on how to remove it from their devices. Alternative or substitute services should also be readily available to users, to ensure uninterrupted continuity of care. If conversational AI was developed to reduce health inequalities, it is vital to consider how these disparities will be addressed through other means in its absence. The impact of conversational AI termination on the health inequalities that it was designed to combat should be carefully monitored and addressed through alternative strategies. It is crucial to devise a comprehensive plan for managing patient data upon the chatbot’s termination and the impact of termination on other systems. Determining the ownership, handling and deletion of patient data is essential, and this information should be well-documented as part of the legal processes surrounding the chatbot’s termination. The responsible handling of patient data is vital for ensuring compliance with data protection regulations and maintaining trust among users and patients. It is also important to gather user feedback on the termination of conversational AI to understand its impact on underserved communities.

## Discussion

The objective of this study was to create a framework for the equitable integration of health-focused conversational AI into healthcare settings. We identified several steps that conversational AI designers and developers should take to minimise potential biases in conversational AI that are related to language and conversational AI expressions. The stakeholders who were interviewed highlighted the importance of involving people from diverse backgrounds, especially those from underrepresented communities, in the co-production, co-development, and collaboration processes while designing, implementing, and discontinuing health chatbots. This community-driven approach to AI is intended to increase inclusivity, acceptability, and engagement, ultimately contributing to the reduction of social health inequalities. The need for clear guidance, regulation, and solid evidence at every stage of conversational AI development and implementation was emphasised, from service conception to maintenance or termination. Although the ten-stage roadmap is based on policy review and in-person interviews, each stage requires a more in-depth exploration of individual activities to ensure conversational AI equity and fairness. Therefore, the roadmap could serve as an essential checklist or guide for health conversational AI developers, particularly those outside of healthcare settings, to help them consider scenarios and activities that can contribute to the effective deployment of conversational AI to tackle health inequalities.

Our findings resonate with a broader discourse in health informatics and AI ethics. The study highlights the necessity of involving individuals from diverse and underrepresented communities in the design and implementation of health conversational AI, akin to the broader participatory design paradigm within health service research, which requires the engagement of end-users and stakeholders to ensure that the technology aligns with the actual needs and preferences of target populations [26,27]. There was also an understanding that the focus of healthcare providers on AI efficiency and cost-effectiveness, without adequate consideration of health equity, is likely to perpetuate algorithmic bias and broaden health inequalities [28]. Our roadmap supports existing literature on various strategies to mitigate bias in machine learning through the adaptation of a systemic approach promoting health equity, from service design to end-stage maintenance [29]. An analysis of 660 documents identified 15 strategies addressing 18 equity issues, such as fostering diversity, improving the quality and quantity of data as well as using equity-focussed checklists, guidelines and tools [30]. Many recommendations of our roadmap support this analysis; however, it also allows conversational AI implementers to recognise and predict specific issues related to individual stages of the conversational AI lifecycle, such as its termination. Thus, our study contributes to the broader scientific literature on methods to improve the impact of AI on health equity, while making them more relevant to conversational AI tools.

Our research highlights the importance of clear guidance, regulation, and evidence at all stages of conversational AI development and implementation. This is further emphasised by the World Health Organization [31], which provides guidelines that governments and regulatory authorities can follow to develop or adapt AI guidance at a national or regional level, ensuring that AI technologies are safely integrated into healthcare systems. Our call for solid evidence at each stage of development aligns with the broader narrative that stresses the need for rigorous evaluation and validation of conversational AI technologies to ensure their safety, effectiveness, and equity both before and after deployment in real-world settings [32,7]. Therefore, due to the potential risk of negative impacts on both individual decision-making and health, including public health outcomes, stemming from inadequately designed and implemented conversational AI in healthcare, our study contributes to the discussion on the ethical challenges of conversational AI. We add our voice to the call for an open science framework and transparency addressing bias in conversational AI for healthcare [33].

### Strengths and limitations

As far as we know, this is the first roadmap that conversational AI developers can follow to ensure that their conversational AI are designed for fairness and equity in the healthcare context. Our methodology involved a combination of content analysis of existing AI policies and framework analysis of interview data through stakeholder consultation. This approach provides a more comprehensive understanding of conversational AI design and implementation, incorporating both existing policies and real-world stakeholder insights from a diverse group of experts and community members.

While the range of perspectives we collected on the equity of conversational AI offered valuable insights into the complexities of conversational AI design and healthcare integration, some of the views were contradictory, particularly regarding the time and resources needed to complete all roadmap stages. Therefore, we acknowledge that specific guidance on our recommended activities is absent and subject to individual interpretation. During our stakeholder consultations, we collaborated with global experts, including those from the United Kingdom and the United States, two countries with distinct healthcare systems. As a result, some aspects of our roadmap may not be entirely relevant or applicable to both contexts. Nonetheless, we are confident that the core principles guiding our roadmap remain valuable for developers across various countries and healthcare systems. Further research is required to establish the optimal scope of these activities and their impact on bias mitigation.

Our study had limitations concerning the varying knowledge levels among participants regarding certain areas of the roadmap. Some did not have enough familiarity with the topic to provide detailed insights, such as aspects related to conversational AI discontinuation in healthcare settings, but they still gave valuable perspectives based on their personal experiences. As such, some expert opinions were hypothetical in their nature in response to various AI implementation scenarios. As the data collection took place in the early months of 2023, there was a considerable difference in stakeholders’ understanding of AI conversational AI, due to the emergence of large language models, such as ChatGPT. As a result, there was significant heterogeneity in responses about conversational AI implementation in healthcare, owing to various perspectives and experiences with conversational AI. There is a chance that the rapid advancements in large language models and their fitness for application into healthcare would require a consideration of different activities to ensure their fairness and equity.

As the design of conversational AI tools for health becomes increasingly prevalent, it is of paramount importance that developers and healthcare providers keep equity, fairness, and patient safety at the forefront of their strategies. Nevertheless, it is also important to understand that health conversational AI might not be the best tool to address health disparities. The causes of specific health inequalities vary, and developers need to comprehend these underlying factors to determine whether their conversational AI could be beneficial or harmful to healthcare settings. To achieve successful development and implementation, it is essential to recognise the limitations and potential applicability of conversational AI for underserved groups. Conversational AI developers need to consider aspects related to access to adequate AI-powered technology, individual cost as well as digital and AI literacy for their users [34]. As this technology continues to evolve, it is highly likely that our roadmap will undergo further development and refinement in the future. As such, it is crucial to establish stronger links between conversational AI designers and diverse communities, patient groups, industry experts, researchers, health professionals, regulators, as well as policy-makers to better inform the direction of conversational AI development and its application. A clear impact of AI technologies on health inequalities needs to be measured and evidenced.

The roadmap provides valuable guidance for ensuring fairness and reducing bias when implementing conversational AI in healthcare. However, practical challenges may arise in applying these principles across diverse real-world contexts. Healthcare systems vary greatly between countries in structure, funding models, regulations, and patient populations [35]. As such, certain activities like extensive user involvement and conversational AI co-production may be difficult to execute fully in resource-constrained settings [36]. The roadmap also recommends close collaboration between developers and healthcare organizations, which may prove challenging where tech expertise is limited. Piloting and incremental implementation would allow for adjustments based on each healthcare system’s unique needs and constraints. Overall, while the guiding principles transcend contexts, flexibility and adaptation of the roadmap’s activities are needed to make conversational AI maximally fair and beneficial across varying healthcare ecosystems globally. Unlike previous frameworks for digital health equity [37] that comprehensively outline various levels of factors affecting digital fairness, our roadmap considers aspects unique to conversational AI.

In conclusion, fostering a culture of transparency, shared learning and centralising resources for easy access to up-to-date industry guidelines and research evidence are vital steps. These efforts would greatly assist health conversational AI developers and healthcare organisations in streamlining their work and enhancing the efficacy and impact of conversational AI. The collaborative approach is crucial in navigating the rapid evolution of AI technologies. With guidance from communities that design, train, and supervise these tools conversational AI can help overcome biases related to healthcare delivery and utilisation instead of perpetuating them. This has the potential to improve patient outcomes and address health inequalities.

## Supporting information

S1 Table List of guidelines with sources used to formulate the framework for stakeholder consultation. (DOCX)

S2 Table Framework for stakeholder consultation. (DOCX)

S3 Table Sample characteristics. (DOCX)
