# Supplementary material for: Achieving health equity through conversational AI: A roadmap for design and implementation of inclusive chatbots in healthcare
Source: PLOS Digit Health. 2024 May 2;3(5):e0000492. doi: 10.1371/journal.pdig.0000492 (PMC11065243; doi:10.1371/journal.pdig.0000492)
Supplement: S1 Table — (DOCX) [file pdig.0000492.s001.docx]

**S1 Table: List of guidelines with sources used to formulate the framework for stakeholder consultation**

| **Name of the guideline/framework** | **Source:** |
| --- | --- |
| Conversational AI, the key to faster and better services for the entire healthcare continuum | <https://f.hubspotusercontent20.net/hubfs/6401175/content/july%20newsletter/Transforming%20the%20Healthcare%20Experience.pdf> |
| Clinical AI Governance | <https://www.babylonhealth.com/en-gb/responsibility/clinical-ai-governance> |
| Chatbots RESET A Framework for Governing Responsible Use of Conversational AI in Healthcare | <https://www3.weforum.org/docs/WEF_Governance_of_Chatbots_in_Healthcare_2020.pdf> |
| Artificial intelligence in healthcare | <https://www.europarl.europa.eu/RegData/etudes/STUD/2022/729512/EPRS_STU(2022)729512_EN.pdf> |
| Guidelines and quality criteria for artificial intelligence-based prediction models in healthcare: a scoping review | <https://www.nature.com/articles/s41746-021-00549-7> |
| Evaluation framework to guide implementation of AI systems into healthcare settings | <https://pubmed.ncbi.nlm.nih.gov/34642177/> |
| Digital, Artificial Intelligence and Robotics Technologies in Education (DART-Ed) | <https://www.hee.nhs.uk/news-blogs-events/news/health-education-england-publishes-roadmap-use-ai-nhs> |
| AI and Healthcare | <https://researchbriefings.files.parliament.uk/documents/POST-PN-0637/POST-PN-0637.pdf> |
| Ethics Guidelines for Trustworthy AI | <https://ec.europa.eu/futurium/en/ai-alliance-consultation.1.html> |
| Ethics and governance of artificial intelligence for health | <https://www.who.int/publications/i/item/9789240029200> |
| Artificial Intelligence: How to get it right | <https://transform.england.nhs.uk/media/documents/NHSX_AI_report.pdf> |
| Harnessing the potential of automation and AI in health care | <https://www.health.org.uk/news-and-comment/blogs/harnessing-the-potential-of-automation-and-ai-in-health-care> |
| A guide to good practice for digital and data-driven health technologies | <https://www.gov.uk/government/publications/code-of-conduct-for-data-driven-health-and-care-technology/initial-code-of-conduct-for-data-driven-health-and-care-technology> |
| Artificial Intelligence in Health and Care Award - Guidance for Competition 3 All Phases | <https://www.nihr.ac.uk/documents/artificial-intelligence-in-health-and-care-award-guidance-for-competition-2-all-phases-stage-1/26004> |
| Advancing AI in the NHS | <http://www.levtankelevitch.com/images/Advancing_AI_in_the_NHS_Polygeia_2018.pdf> |
| Thinking on its own: ai in the nhs | <https://reform.uk/research/thinking-its-own-ai-nhs> |
| Framework for evaluating the safety, acceptability and efficacy of AI systems for health (NIHR) | <https://arxiv.org/abs/2104.06910> |
