# Supplementary material for: Achieving health equity through conversational AI: A roadmap for design and implementation of inclusive chatbots in healthcare
Source: PLOS Digit Health. 2024 May 2;3(5):e0000492. doi: 10.1371/journal.pdig.0000492 (PMC11065243; doi:10.1371/journal.pdig.0000492)
Supplement: S2 Table — (DOCX) [file pdig.0000492.s002.docx]

**S2 Table: Framework for stakeholder consultation**

| **Patient-facing AI health chatbot implementation** | |
| --- | --- |
| **Design phase** | Needs assessment for health chatbot |
|  | Develop evidence base for health chatbot, |
|  | Review up-to-date guidelines regarding AI and chatbots in healthcare |
|  | Define chatbot role/clinical and administrative tasks |
|  | Reduce complexity and increase ease of use |
|  | Select appropriate patient-facing platform/IT system |
|  | Fully readable and shareable datasets for external data quality checks; build interoperability of systems |
|  | Develop and optimise content pathways for comprehension, literacy and interactivity |
|  | Define relevant patient groups and their characteristics |
|  | Define underlying behaviour change framework |
|  | Adopt user-centred approach |
|  | Collaborative design/stakeholder involvement and chatbot co-production |
|  | Reduce bias, introduce equity measures, diversity & inclusion in chatbot design and deployment |
|  | Build in patient safety measures, safeguarding measures, human contact pathways |
|  | Define chatbot outcomes |
|  | Proof-of-concept testing |
|  | Assess chatbot language accuracy via user testing |
|  | Optimise chatbot language, translation checks and adjust medical terminology |
|  | Provide access to AI training datasets |
| **Pre-implementation phase** | Define adequate level of governance (i.e. information governance); gain regulatory requirements and approvals |
|  | Define data use, storage, access, privacy settings and encryption |
|  | Optimise confidentiality and cybersecurity |
|  | Assess stakeholder acceptability, trust and address hesitancy |
|  | Develop chatbot transparency & explanability |
|  | Explore and address ethical issues |
|  | Develop reporting of adverse events and unintended consequences |
|  | Develop staff training materials and resources on how to interact with chatbots |
|  | Clarify outstanding legal and licencing matters (i.e. intellectual property, third part involvement) |
|  | Accountability for system failure |
|  | Assess feasibility via simulation research and/or pilot study with specific patient groups |
|  | Assess patient usage, satisfaction and confidence in chatbot advice |
|  | Measure impact on behaviour change and 'soft outcomes' |
| **Implementation phase** | Optimise administration and supervision |
|  | Select chatbot champions to overlook its deployment |
|  | Assess uptake and continued chatbot use as part of digital healthcare service |
|  | Monitor utilisation across various patient groups |
|  | Set-up regular auditing |
|  | Measure impact on healthcare services and clinical team workload |
|  | Chatbot fidelity |
|  | Sustainability |
|  | Evaluate cost |
|  | Service evaluation with multiple outcomes (i.e. pre/post design, comparative design) |
|  | Complaint management |
|  | Demonstrate benefits and costs of chatbot implementation |
| **Post-implementation phase** | Scalability and generalisability of the chatbot across trusts and the entire NHS |
|  | Integration & tailoring, define local variations in chatbots - level of standardisation |
|  | Comparative evaluation (RCT design) |
|  | Assess cost-effectiveness |
|  | Impact on healthcare environment |
|  | Chatbot termination procedures |
|  | Plan for system updates and technical improvements |
|  | Raising awareness and chatbot promotion |
