# Supplementary material for: Achieving health equity through conversational AI: A roadmap for design and implementation of inclusive chatbots in healthcare
Source: PLOS Digit Health. 2024 May 2;3(5):e0000492. doi: 10.1371/journal.pdig.0000492 (PMC11065243; doi:10.1371/journal.pdig.0000492)
Supplement: S3 Table — (DOCX) [file pdig.0000492.s003.docx]

**S3 Table: Sample characteristics**

**Industry experts**

**Gender Ethnicity Role**

P1 Male White British Technical expert chatbot developer

P2 Male White British NHS doctor in sexual health

P3 Female White US Academic/sexual & reproductive health chatbot

P4 Male Black African Developer/sexual & reproductive health chatbot

P5 Female White US Academic/sexual & reproductive health chatbot

P6 Female White US 3^rd^ sector/sexual & reproductive health chatbot

P7 Female White European 3^rd^ sector/sexual & reproductive health rights

P8a Male Asian Indian Technical expert chatbot developer

P8b Female Asian Indian 3^rd^ sector/sexual & reproductive health chatbot

P9 Male White British Academic/sexual & reproductive health chatbot

P10 Male Asian Chinese Doctor/symptom checker chatbot

P11 Female Northern Irish 3^rd^ sector/mental health chatbot

P12 Female Asian Chinese Academic/sexual & reproductive health chatbot

P13 Male Northern Irish 3^rd^ sector/mental health chatbot

P14 Male White US Academic/sexual & reproductive health chatbot

P15 Male Middle Eastern Doctor/specialist in AI & robotics

P16 Male Latin American Developer/mental health chatbot

P17 Female Northern Irish Academic/mental health chatbot

P18 Male White European Developer/chatbot life coach

P19 Female White British NHS doctor in sexual health

P20 Male White British NHS doctor & digital lead

P21 Female Asian Chinese NHS doctor/academic & equitable AI specialist

P22 Female White British NHS mental health nurse/clinical safety officer

P23 Female White British Developer/NHS signposting chatbot

**Community members**

**Gender Ethnicity Age­­­­­­­­­­­­­­­­­­­­­­­ Sexual orientation**

P24 Male Asian Chinese 21 heterosexual

P25 Female White Other 30 heterosexual

P26 Female Black Mixed 23 heterosexual

P27 Female Latin American 25 heterosexual

P28 Male Latin American 29 gay

P29 Female Black African 33 heterosexual

P30 Female Black African 55 heterosexual

P31 Female Black African 26 heterosexual

P32 Female Asian Bangladeshi 20 heterosexual

P33 Male Black African 27 heterosexual
